# The neural development of empathy is sensitive to caregiving and early trauma

**DOI:** 10.1038/s41467-019-09927-y

**Published:** 2019-04-23

**Authors:** Jonathan Levy, Abraham Goldstein, Ruth Feldman

**Affiliations:** 1 Interdisciplinary Center, Herzliya, 46150 Israel; 2 Gonda Multidisciplinary Brain Research Center and Department of Psychology, Bar-Ilan University, Ramat Gan, 5290002 Israel; 3 Yale University, Child Study Center, New Haven, CT 06520 USA

**Keywords:** Neuroscience, Empathy

## Abstract

Empathy is a core human social ability shaped by biological dispositions and caregiving experiences; yet the mechanisms sustaining maturation of the neural basis of empathy are unknown. Here, we followed eighty-four children, including 42 exposed to chronic war-related adversity, across the first decade of life, and assessed parenting, child temperament, and anxiety disorders as contributors to the neural development of empathy. At preadolescence, participants underwent magenetoencephalography while observing others’ distress. Preadolescents show a widely-distributed response in structures implicating the overlap of affective (automatic) and cognitive (higher-order) empathy, which is predicted by mother-child synchrony across childhood. Only temperamentally reactive young children growing in chronic adversity, particularly those who later develop anxiety disorders, display additional engagement of neural nodes possibly reflecting hyper-mentalizing and ruminations over the distressing stimuli. These findings demonstrate how caregiving patterns fostering interpersonal resonance, reactive temperament, and chronic adversity combine across early development to shape the human empathic brain.

## Abstract (web summary)

People’s early experiences and dispositions influence their ability to show and feel empathy. Here, using a sample of children exposed to war-related trauma, the authors examine how parenting, temperament, anxiety, and adversity affect the maturation of neural responses associated with empathy.

## Introduction

Empathy, the capacity to resonate with and reflect upon the feelings and mental states of others^1^, is a core social ability sculpted by a long history of mammalian evolution to enhance species survival, afford group communication, and enable social life. To tap the neural mechanisms and maturational process of empathy, research has employed a cross-species approach and demonstrated that both rodents and nonhuman primates exhibit rudimentary empathy expressed in contagion and mimicry^2^. Complementing this effort, neuroimaging studies in humans, typically targeting the adult brain, show that while human empathy similarly implicates automatic resonance to others’ distress, it also integrates higher-order neural activations that afford mentalization of others’ feelings and generation of an empathic response that differentiates self from other^3^. Yet, to understand the unfolding of human empathy from its origins, research must complement the phylogenetic approach with an ontogenetic one by utilizing prospective longitudinal studies that can pinpoint factors which over time facilitate or undermine maturation of the human empathic brain.

Developmental evidence indicates that the capacity for empathy emerges across the first years of life through complex interactions between the child’s biological dispositions and the quality of caregiving^4,5^. In parallel, research in social neuroscience describes dramatic shifts in maturation of the neural systems that sustain empathy^6,7^; yet, the determinants that shape this neural development remain obscure. To track how multiple factors integrate to support maturation of the neural empathic response, we utilized a longitudinal study of children followed from early childhood to preadolescence and focused on three factors known to impact children’s empathic abilities. These include chronic early life stress (ELS), synchronous and attuned parent-child relationship, and temperamental reactivity implying heightened inborn responsivity to negative stimuli. Since prolonged adversity, particularly exposure to ELS, negatively impacts various social functions^8–10^ including empathy^11–15^, we followed a cohort living in a distinct ELS context, repeatedly observed mother–child interactions in the home environment, assessed temperamental reactivity in early childhood, and evaluated children’s anxiety disorders in late childhood to test their direct and indirect effects on the neural substrates of empathy at the transition to adolescence.

The daily experience of empathy involves both simulation of the bodily and affective states of others and drawing inferences about their mental states^1,16,17^. Yet, the early studies on the neuroscience of empathy distinguished between these two processes and examined them separately under laboratory conditions. Subsequently, conceptual models differentiated two components of empathy; affective empathy/resonance and cognitive empathy/mentalization^1,17^, a dichotomy that was mapped into distinct brain structures and neural networks. The first, affective empathy/resonance, was thought to involve automatic response to others’ pain and feelings and to rely on structures that support sensorimotor perception and their representation in one’s own brain, such as the primary somatosensory and motor cortices, anterior cingulate cortex, and sensorimotor area (SMA), implicating the embodied simulation network; the second, cognitive empathy/mentalization integrates higher-order cortical regions to understand others’ mental life and includes the prefrontal cortex (PFC), temporoparietal junction, superior temporal sulcus (STS), and temporal pole, which comprise the mentalizing network^1,2,17^.

**Fig. 1:**
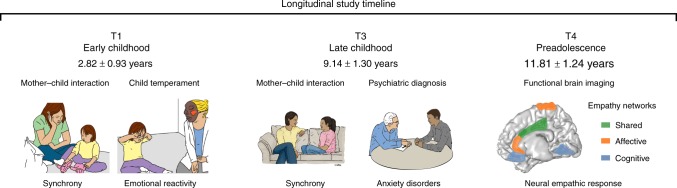
Longitudinal study timeline. In T1, at early childhood, child and mother interacted with each other; mother–child synchrony was calculated. In T3*, Psychiatric diagnosis was conducted and child and mother interacted again and the synchrony construct was again computed; hence Maternal Synchrony scores leaned on the T1 and T3 interactions. In T4, neural empathic response to vicarious distress was evaluated using MEG neuroimaging. Illustration of empathy networks is inspired by the comprehensive review of de Waal and Preston^2^. *The Synchrony illustration is adapted and reproduced by permission of Oxford University Press. ^©^ The Author 2017. All rights reserved. For permissions, please email journals.permissions@oup.com. This figure is not included under the open access license of this publication. The original figure is Fig. 1a—Jonathan Levy, Abraham Goldstein, Ruth Feldman, Perception of social synchrony induces mother–child gamma coupling in the social brain. *Soc. Cogn. Affect. Neurosci.* 2017; 12 (7): 1036–1046

Notwithstanding this distinct cerebral mapping (Fig. 1, right panel), such dual dissociation model is somewhat artificial. Drawing parallels between this and other dual models^18^, in real-life situations, one typically employs both processes, albeit to varying degrees pending on person and context. Indeed, ecologically valid experiments that simulate real-life settings indicate that the two networks reflect two facets of social living and function in concert to support human empathy^10,16,17,19^. One important observation from these studies was that under natural settings, the sensorimotor area and the middle cingulate cortex (SMA/MCC) underpin both embodied simulation and mentalizing processes, integrating the affective and cognitive components of empathy^2,20,21^ (Fig. 1, right panel in green). For instance, an empathy paradigm that exposed participants to distressing stimuli of everyday life (triggering embodied simulation) while asking participants to take the target’s perspective (activating mentalizing) yielded activations containing the SMA/MCC node^22^. Here, we adopt the same paradigm to probe empathy (hereafter we use the term “empathy” for the two facets, unless otherwise specified) and test the developmental precursors of this shared empathy network.

Despite growing interest in the neuroscience of empathy, there are nearly no data on the developmental processes that tune the brain toward an empathic response. Several factors may contribute to neural empathy, the first is sensitive caregiving. In particular, the experience of parent-child synchrony, the parent’s ongoing resonance and online adaptation to the child’s nonverbal signals and verbal communications, is associated with children’s empathy across childhood and adolescence^23^. The mother–child bond provides a setting where synchrony is first experienced and encoded in the brain^24^, creating a template for the child’s later resonance with the distress, feelings, and thoughts of others^25^. When the mother’s capacity to provide synchronous parenting is compromised, for instance, in cases of postpartum depression, children show reduced empathic behavior^26^ and impaired neural empathic response to others’ pain in adolescence^27^. Synchrony is the process by which mother’s brain impacts the child’s brain and wires it to social participation^25^; during moments of behavioral synchrony mother and child’s brains synchronize in the STS^28^, a social neural hub, and behavioral synchrony across the first 6 years predicts adolescents’ neural response to attachment cues in key nodes of the social brain, including the STS, STG, and Insula^29^.

Second, exposure to chronic adversity impairs social–emotional processing^4,11–15^ and the effect is most prominent when adversity begins early and persists throughout early childhood^9,30,31^. Research utilizing fMRI^13^ and MEG^11^ show that trauma alters neural responses that underpin the affective and cognitive components of empathy. Adults exposed to early trauma exhibit abnormal neural response to negative emotional stimuli and impairments in the brain basis of social functions^30,32^, and children and adolescents exposed to ELS display impaired processing of affective facial expressions^33,34^, implying disruptions to emotional processing which sustains empathy. Thus, while no direct evidence links ELS to children’s neural empathic response, these lines of research lend support to this hypothesis.

Although chronic ELS disrupts social functioning, substantial individual differences exist, which are shaped by biological dispositions as they interact with the specific adversity^35^. Most studies on the long-term effects of ELS employed biology-by-context models that target variations in dispositional stress reactivity as they interact with stressful environments^36^. Heightened stress reactivity reflects increased biological sensitivity to context, which augments the effects of stress on negative outcomes under conditions of ELS^37,38^. One mechanism proposed to mediate the detrimental effects of ELS on social outcome is temperamental reactivity, the heightened dispositional response to negative stimuli. Early adversity augments attention to negative and frightening events^31^, and when combined with inborn reactivity to negative stimuli, it may lead to difficulties in disengaging from distressing cues^39^. Such exaggerated response is often accompanied by repeated mentalization and ruminations over the distressful event^40^. Moreover, early temperamental reactivity increases the propensity to develop anxiety disorders in later childhood and adolescence^41^. Anxiety disorders, in turn, impair the neural basis of multiple social–emotional functions^42^, are associated with increased ruminations over negative events, and link with inability to disengage from negative stimuli^43^.

The current decade-long prospective longitudinal study integrated repeated observations of parenting with lab-based assessment of negative reactivity and psychiatric evaluations to predict the neural basis of empathy among children exposed to ELS versus controls. We utilized a unique cohort of children exposed to war-related trauma since birth who experience frequent, unpredictable exacerbations of the traumatic situation. Children and their families were followed from early childhood to early adolescence (Fig. 1) and thus, our study affords a rare “natural experiment” in ELS research that typically includes heterogeneous adversities. In preadolescence (11–13 years), we used magnetoencephalography (MEG) to probe children’s oscillatory response to others’ distress and focused on alpha rhythms, which underpin empathic processes^44,45^ and express in children as late alpha-band enhancement in sensory cortex to others’ pain^7^. Here, our paradigm additionally involved perspective-taking and was expected to activate substrates supporting both affective and cognitive empathy, such as the SMA and MCC^2,20,21^.

We formulated two hypotheses and one open research question. First, we expected that the neural empathic response in preadolescence will implicate structures that support the shared affective and cognitive empathy, namely the SMA and the MCC, and that these activations will be underpinned by late alpha-band enhancement. Second, we hypothesized that activation of this neural network will be longitudinally predicted by mother–child synchrony across the first decade of life. Next, since no prior research linked ELS with the neural development of empathy, we explored direct and indirect ways by which ELS impacts the neural empathic response as an open research question. For this goal, we explored neural patterns that are specific to trauma-exposed youth and tested their associations with temperamental reactivity and anxiety disorders. Consistent with the biological sensitivity to context model^37^, we expected that only among trauma-exposed youth, these putative neural patterns will be shaped by early reactivity. Additionally, we conjectured that these stress-specific activations would link with the consolidation of a distinct anxiety disorder in late childhood. We find that at preadolescence, the neural empathic response implicates structures tapping the overlap of cognitive and affective empathy, such as SMA and MCC. In addition, adolescents’ neural empathic response is sensitive to caregiving across the first decade, particularly mother–child synchrony, and to chronic early trauma. Finally, temperamentally reactive children reared in stressful contexts are more likely to develop anxiety disorders and show additional activation in nodes possibly reflecting hyper-mentalizing and difficulties in disengaging from negative cues.

## Results

### Group differences in study variables

A tendency toward lower (*t* = −2.29, *P* = 0.02_uncorrected_, *t* test; Cohen’s *d* = 0.50; 95% confidence interval [0.056–0.800]) mother–child synchrony was found for war-exposed families (*M* = 3.45, SD = 0.90) compared to controls (*M* = 3.88, SD = 0.78). No statistically significant differences (*t* = −0.95, *P* = 0.34, *t* test) emerged in temperamental reactivity between exposed (*M* = 0.37, SD = 0.09), and control (*M* = 0.35, SD = 0.12) children. War-exposed children were significantly more likely to develop an anxiety disorders (50%), compared to only 11.90% among controls, *Χ*^2^(1) = 14.03, *P*_FDR-cor_ = 0.0002, *Χ*^2^-test; Cramer’s *V* = 0.41. Comparing the current sample (*N* = 84) and those lost to attrition from T1 (*N* = 148), there were no significant differences on synchrony and temperamental reactivity (*t* = 1.50 and −0.48, *P* = 0.13 and *P* = 0.63, respectively, *t* tests).

### Self-reported empathy

To provide validation for the imaging paradigm, participants rated the ease of perspective-taking during the empathy paradigm on a five-point Likert scale (1-very difficult-5-very easy). Children were generally able to take the perspective of the protagonist (*M* = 3.82, SD = 0.76). Participants also rated the emotions conveyed in the paradigm by rating the affective valence (1-very negative-5-very positive) and arousal (1-very low-5-very high) of the stimuli. Participants rated the stimuli as displaying negative affect (*M* = 1.90, SD = 0.60) and high arousal (*M* = 4.15, SD = 0.79). These findings provide a validation check that the current stimuli were perceived by the children as highly distressing and that they had no difficulty taking the perspective of the protagonist in distress. There were no group differences in these three measures (*t* = 0.84, *t* = 0.44, and *t* = −1.51, *P* = 0.40, *P* = 0.66 and *P* = 0.13, respectively, *t* tests).

### MEG results

**Fig. 2:**
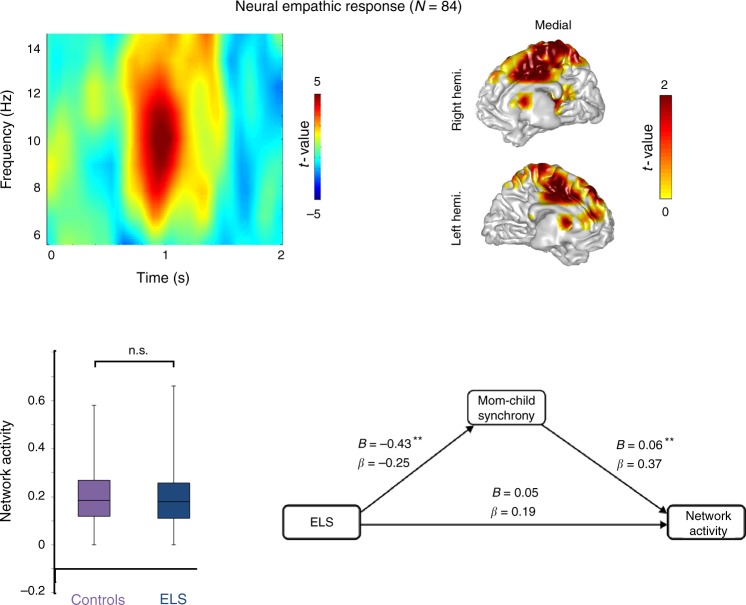
Neural empathic response. Preadolescents activated the SMA/MCC (right upper panel) in response to vicarious distress *(*masked for *P*_cluster-cor_ < 0.05), and the color bar represents masked significant *t* values. This activation was expressed as late alpha-band enhancement (left upper panel). The red blob represents the significant time-frequency window (*P*_cluster-cor_ < 0.05), and the color bar conveys the *t* values. This neural effect of empathy was not significantly different between the two groups (left lower panel), yet mother–child synchrony in early and late childhood mediated (**P_FDR-cor_ < 0.05) the effects of trauma exposure on this neural effect (right lower panel). In the chart bars, points are laid over a SEM (95% confidence interval)

We proceeded to test our two hypotheses: First, we contrasted stimuli involving distress (DS) versus no distress (no-DS). The statistical time-frequency contrast (0–2 s; 6–14 Hz) of all MEG sensor-array is represented in Fig. 2 (left upper panel), with significant time–frequency patterns (*P*_cluster-cor_ < 0.05, permutation test): children exhibited greater alpha-band enhancement (7–12 Hz) at 800–1200 ms above anterior-central sensors. Source localization (masked at *P*_cluster-cor_ < 0.05, permutation test) revealed that the alpha oscillatory pattern emanated mainly from the SMA and MCC, the shared network of affective and cognitive empathy (Fig. 2, right upper panel), supporting our first hypothesis. Second, to explore whether ELS directly impacts this neural response, we compared the two groups; however, no statistically significant difference emerged (*P* = 0.34, *t* test) (Fig. 2, left lower panel). This finding demonstrates the ELS per se does not directly impact preadolescents’ neural empathic response.

**Fig. 3:**
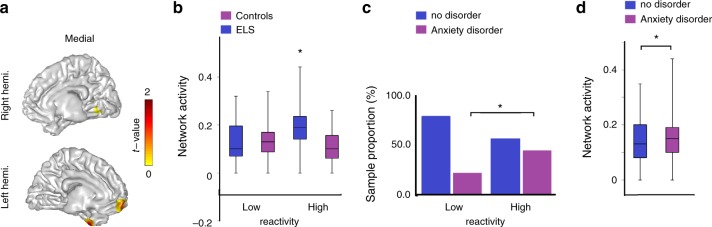
Characterizing the Putative Mentalizing activity. **a** ELS preadolescents activated the putative mentalizing network in T4 significantly stronger than controls did (masked for *P*_cluster-cor_ < 0.05), and the color bar conveys masked *t* values. **b** Power averages over the network nodes were significantly different between the two groups, particularly those with high negative reactivity in T1. **c** Individuals with high negative reactivity in T1 developed more internalizing disorders in T3. **d** Internalizing disorders in T3 explained that mentalizing neural activity, in T4. In the chart bars, points are laid over a SEM (95% confidence interval), and asterisks convey statistically significant effects

We proceeded to our open question and probed the effects of ELS on neural activations to others’ distress by contrasting between groups at the whole brain level in search for cerebral nodes that may show group differences. This contrast yielded greater activations only for the war-exposed group in the following regions: vmPFC, fusiform gyrus, and temporal gyri, while correcting for whole-brain voxels (*P*_cluster-cor_ < 0.05, permutation test) (Fig. 3a). These nodes have been observed in mentalizing/cognitive empathy tasks that are not shared with embodied simulation tasks; thus, it is reasonable to consider this activation as related to this process, although such inference cannot be directly tested. For consistency with prior studies, we hereafter label this activation pattern as mentalizing activation and elaborate on this labeling in the “Discussion”. Averaging activation values in this network’s nodes yielded a statistically significant difference (*P*_FDR-cor_ = 0.008, *t* test; Cohen’s *d* = 0.60; 95% confidence interval [−0.093 to −0.015]) between the two groups (*M* = 0.03, SD = 0.10 and *M* = −0.02, SD = 0.07 in the war-exposed and controls, respectively).

### Brain and behavior

We then proceeded to test whether the empathic neural response or the selective exposed-group activation (i.e., mentalizing) correlated with the behavioral, developmental, and psychiatric measures. For this goal, we averaged power values in the different nodes of each network. To verify whether there was any link between the neural and the self-reported measures of empathy, we computed Pearson’s correlations (Supplementary Table 1) and this did not yield any statistically significant results (*P* > 0.08). Following, to further test our second hypothesis and open question, we examined the associations between the neural data with the observed longitudinal variables (mother–child synchrony, child temperamental reactivity, and anxiety disorders) first by Pearson’s correlations (Supplementary Table 1) and then by two hierarchical regression models (Table 1). As seen, the empathic neural response was independently predicted by mother–child synchrony across the first decade, while the mentalizing network response was explained by trauma exposure and by the child’s reactive temperament and marginally by the presence of an anxiety disorder. In addition, mediation analysis showed that mother–child synchrony mediated (*P*_FDR-cor_ < 0.05) the effects of trauma exposure on the neural basis of empathy (Fig. 2, lower panel), thereby confirming our second hypothesis by charting an indirect link between ELS and empathic response.

**Table 1:** Hierarchical linear regression predicting neural response from first decade variables

	Empathy response			Mentalizing/reactivity		
Predictors	Beta	*R*^2^ change	FChange	Beta	*R*^2^ change	Fchange
Exposure	0.14	0.004	0.26	0.18	0.083	6.68^*^
Synchrony	0.38	0.109	8.94**	−0.06	0.001	0.056
Reactivity	−0.19	0.032	4.427	0.12	0.049	4.15^*^
Anxiety disorders	0.06	0.004	0.28	0.21	0.041	3.55^+^

For the empathy columns, *R*^2^ total = 0.15 (*F* (4, 76) = 3.07, *p* < 0.02) whereas for the mentalizing/reactivity columns *R*^2^ total = 0.17 (*F* (4, 76) = 3.64, *p* < 0.01). *p < 0.05, +*p* = 0.06

Finally, to complete the exploration on the impact of ELS on the neural empathic response, we conducted three additional analyses: (a) univariate analysis of variance examined the effects of ELS and negative reactivity (high/low using the median split. Median = 0.34) on the mentalizing network. (b) *Χ*^2^ test examined the relation between negative reactivity and anxiety disorders. (c) One-way analysis of variance tested whether participants with anxiety disorders activated the mentalizing network more than those without internalizing disorders. Results showed main effects for ELS (*F*(1,78) = 6.49, *P*_FDR-cor_ = 0.013, one-way ANOVA), main effect for negative reactivity (*F*(1,78) = 7.48, *P*_FDR-cor_ = 0.008, one-way ANOVA), and an interaction of the two (*F*(1,78) = 8.96, *P*_FDR-cor_ = 0.004, one-way ANOVA). Only among trauma-exposed youth, those with higher temperamental reactivity exhibited significantly more of the network’s activation (i.e., mentalizing) compared to children with low reactivity (*t* = −4.81, *P*_FDR-cor_ = 0.00002, *t* test), but no differences emerged between high- and low-reactive control children (*t* = −0.28, *P* = 0.42, *t* test) (Fig. 3b), findings consistent with the biological sensitivity to context model. Further, highly reactive children were significantly more likely to develop anxiety disorder (44.12%), compared to only 21.42% among low-reactive children, *Χ*^2^(1) = 4.48, *P*_FDR-cor_ = 0.03, *Χ*^2^-test; Cramer’s *V* = 0.24 (Fig. 3c). Finally, children with anxiety disorder developed more robust (*F*(1,80) = 6.36, *P*_FDR-cor_ = 0.01, one way ANOVA) neural activity in the mentalizing network, compared to those without disorder (Fig. 3d). Hence, greater temperamental negative reactivity to stress at T1 largely predicted the development of anxiety disorder in T3, and both largely explained the neural response of nodes in the mentalizing network at T4.

## Discussion

Empathy is a core social ability that enables humans to resonate with the pain, distress, affect, and mental states of others and maturation of this ability has been described across evolution in rodents, nonhuman primates, and humans^1,2,46,47^. Our prospective longitudinal study addresses the ways by which positive and negative experiences across the first decade of life shape the neural basis of empathy in human children. Following families from early childhood to adolescence and integrating scientific methodologies from diverse fields including developmental psychology, psychiatry, and social neuroscience, we test how chronic adversity, parent–child synchrony, temperamental reactivity, and anxiety disorders jointly shape the neural empathic response at the transition to adolescence. We target the two components of empathy as a unitary phenomenon that integrates automatic and representational aspects and employ a paradigm that simulates the two facets of empathy^22^, affective/resonance and cognitive/mentalizing. Results indicate that preadolescents respond to others’ everyday distress by activating the shared affective and cognitive empathy nodes, the SMA and MCC, and their neural empathic response is accompanied by self-reported recognition of the protagonist’s distress and ease at taking his/her perspective. Our findings further suggest that this neural response marks a distinct social neural process, not mere reactivity to negative emotional stimuli, as no associations emerged between child negative reactivity and the neural empathic response. Moreover, parent–child synchrony across the first decade, the experience of interpersonal resonance within the mother–child attachment, longitudinally shapes the neural basis of empathy in adolescence.

How does chronic early stress impact the neural basis of empathy? It appears that ELS does not have a direct effect on the shared neural empathic response but exerts several indirect effects, which increase the likelihood of altered response for some children, but not for others. Our findings indicate that some children are more vulnerable to the consequences of early trauma and chart two pathways by which stress exerts its influence on the empathic brain, relational path and biological path. First, trauma-exposed mothers and children engage in less synchronous interactions and the reduced synchrony mediates the link between ELS and attenuated neural empathic response; hence, behavioral synchrony charts a key relational pathway by which the child’s brain is tuned to social life. Second, stress-exposed children activate additional nodes distinctly implicated in the mentalizing network, not the shared empathic network, and these activations are not observed in control children. A closer look reveals that these trauma-specific mentalizing activations are predicted by the child’s anxiety disorders. This raises the possibility that such mentalizing activity does not reflect empathy per se but indexes emotional reactivity to the distressing stimuli. In fact, this additional neural response at preadolescence is fully moderated by the child’s temperamental reactivity to negative emotions in early childhood and only appears among highly reactive children exposed to chronic stress. The mentalizing-based activation is also explained by the presence of anxiety disorders in late childhood and these disorders are more prevalent among children born with more reactive temperament^41^. We suggest that these added activations, found among highly reactive children reared in stressful contexts and particularly among those on a more risky trajectory, may represent increased ruminations and inability to disengage from negative stimuli, which characterize adolescents with anxiety disorders^43^.

Exposure to stimuli depicting others in distress, including both physical pain and mental distress, activates the SMA/MCC node^22^, with MEG studies implicating alpha modulations in sensorimotor cortex^7,44,45^. Our findings add ecological-validity to this literature by showing that this neural pattern is triggered by age-appropriate daily distress of peers, and our use of MEG demonstrates alpha modulations over sensorimotor regions, similar to findings for pain empathy and motor mirroring, suggesting that complex resonance with others’ mental states builds on the same circuit. As activations nested in the SMA and MCC are proposed to sustain both aspects of empathy, cognition and affect^2,20^, our findings suggest that although these nodes are a core part of the embodied simulation system that enables individuals to represent others’ motions and emotions in their own brain and initiate an automatic, bottom-up resonance, they also have a top-down regulatory role in conveying more abstract representations that sustain empathy. Consistently, Fan et al.’s^20^ review of neuroimaging studies on empathy shows that the SMA-MCC node is repeatedly activated across a wide variety of empathy studies, regardless of paradigm, stimulus, or emotion, including pain, fear, happiness, disgust, and anxiety.

The capacity for empathy is critical for the human ability to participate in social life, feel compassion, sustain a sense of self, and form affiliative bonds, and like any other core social ability develops in mammals in the context of the mother–infant bond^24,25^. Parent–child synchrony provides the first experience of nonverbal resonance where the mother adapts her gaze, affective expression, vocal quality, and movements to the infant’s earliest signals to create a shared dialog. Synchrony supports the development of abilities that sustain social engagement, including symbol formation, moral understanding, emotion regulation, and frustration tolerance and provides a template for biological synchrony; during synchronous moments parent and child coordinate their heart rhythms^48^, neural response^28^, and oxytocin release^49^, hence, synchrony is a mechanism by which the parent’s mature physiological systems externally-regulate the child environment-dependent systems and tune them to social life. Here, we show that synchrony longitudinally shapes the neural basis of empathy in preadolescence and specifically targets brain areas that underpin the interface of cognitive and affective empathy. Because conditions that impair maternal–infant bonding, such as postpartum depression, premature birth, and contextual adversity impinge upon the experience of synchrony, our findings highlight the need to construct early interventions that aim to bolster synchrony in the first years of life.

Empathy is defined as the ability to share the affective and mental state of others^1,2^. Yet, this overlap of self and other may also be considered as depicting emotional reactivity. To dissociate the terms, it is necessary not only to measure neural responses to vicarious states, but also to probe other measures of affect, including social behavior and self-reports. Although the empathy paradigm used here has been previously shown to elicit an empathic neural response^22^, the measures we collected during the pilot and study confirm that preadolescents recognized the vicarious distress expressed in the stimuli and were reasonably able to take the vicarious perspective. Hence, these data suggest that children were empathizing with the targets, that is, they recognized their emotions and were able to take their perspective. In addition to self-reports during the experiment, we also utilized a formal well-known assessment of temperament in early childhood as predictor of emotional reactivity to vicarious distress at preadolescence. We found that such temperamental reactivity did not explain the shared empathic neural response to the stimuli; however, early reactivity was significantly predictive of the neural response that was selective to trauma-exposed youth (i.e., mentalizing). Our findings suggest that, overall, the vicarious stimuli elicited an empathic response, consistent with previous research^22^, but that preadolescents with a history of trauma may additionally develop a negative emotional reaction that should not be interpreted as empathy per se despite its reliance on neural nodes putatively assigned to the cognitive empathy (i.e., mentalizing) network^17^.

The observation that negative emotional reactivity in early childhood predicts both the emergence of anxiety disorders in late childhood and activation of the mentalizing network in preadolescence may be taken as indication of over-mentalization while preadolescents are exposed to others’ distress. However, such interpretation is indirect, requires caution and should be considered in light of previous research. For instance, it has been shown that war-related trauma may alter neural networks underpinning the perception of aversive stimuli in ways that increase higher-order processing compared to automatic sensory processing^8^, and that individuals exposed to war-related trauma present neural abnormalities related to stress and fear regulation^50^. Specifically, damage in core regions of the mentalizing network is associated with diminished empathy in trauma-exposed war veterans^12^. Consistent with the current findings, trauma exposed individuals show hyper-activation of the mPFC during a mentalizing empathy task similar to the task used here^13^. In order to cope with distressing stimuli, war veterans employ compensatory mental strategies^15^, and it is possible that war-exposed children are less competent in downregulating and suppressing the negative thoughts elicited by the presented negative stimuli, consistent with prior research^40^. War-exposed children may identify more closely with the targets in distress, as this network activates when imagining oneself in similar situations^17^. Our findings corroborate other lines of research on the relations between anxiety and augmented attention to distressing stimuli^39^ and over-activation of the PFC during cognitive reappraisal^40^. It is thus possible that the over-activation of these regions in response to distressing stimuli presents a regulatory neural dysfunction that may, in some cases, lead to the increased ruminations, anhedonia, and attention difficulties that underpin anxiety disorders. Consistent with this assumption, studies have shown that ruminations are sustained by enhanced neural activity in the mPFC^51,52^.

Although preadolescents reported feeling negative emotions and perspective-taking during the empathic task, we did not find a one-on-one correspondence between the degree of network activation and the reported emotions and perspective-taking. Such findings are not at odds with many empathy studies, which typically do not attempt to compare neural data and simple self-reports of empathy, and the few studies that compared between the two did not find significant correlations^44,53^. One explanation for this lack of associations is that neural data may be more sensitive to certain unconscious or even conscious empathic processes than self-report questions^54^. Another interpretation is that self-reports of empathy during the paradigm may not tap into the same processes that are mapped by the neural data. For instance, Morelli et al.^22^ used the same paradigm on adults and showed that one specific node in the neural response explained the participants’ daily prosocial behavior, a measure that was collected over several days outside the laboratory and indexed the individual’s habitual, not momentary, empathic style. In the present study, although children’s neural response did not correlate with their self-report during the task, the neural data correlated with measures of mother–child synchrony, a core dyadic process. Moreover, one scale of the synchrony construct in late childhood is behavioral empathy (e.g., “I see why you think this is fun” in the positive discussion; “I understand that leaving my clothes on the floor is really annoying” in the conflict discussion). Hence, the empathic neural response measured here may be more sensitive to empathic social behavior in ecologically valid settings than to self-reported empathy in a laboratory setting.

Despite the significance of our findings, one limitation is that neuroimaging data are available only at preadolescence; hence, it is impossible to test whether the effects reported here convey functional or anatomical shifts during the course of development. Such endeavor would be ambitious and future studies, including form the current cohort, should employ this approach. We recently found major shifts in maturation of the neural rhythms that sustain pain empathy across development, particularly in the alpha rhythm^7^. This suggests that alpha rhythms undergo a major developmental process which may be fundamental, or even critical, for the maturation of empathy and further longitudinal observations are needed to fully understand the mechanisms by which ELS in general and war-related trauma in particular impact the developing brain. Another point to keep in mind in the interpretation of the cortical activations is the limited spatial resolution of MEG sources, especially given the unstable head position in pediatric samples, although this concern is partially mitigated by the large sample-size which increases the statistical power and reliability of the reported findings. Furthermore, although our study affords a unique ‘natural experiment’ of adversity, as adversity was relatively similar across participants, it is possible that individuals may have been exposed somewhat differently to the external events. Previous research showed that different dimensions of exposure, such as proximity to disaster, may impact neural response to stress^55^, and although all our exposed children lived in the same frontline neighborhoods, some subtle differences in exposure may have still impacted their neural response. It is also important to remember that while our study is longitudinal and we use the term prediction in the statistical sense, our findings by no means imply causality and only describe long-term associations between parenting, temperament, early adversity, and brain response. Finally, our study taps a key epistemological issue on the validity of neuroimaging an internal state and the correspondence between the third and first-person perspectives. While this issue is inherent in the neuroscience of empathy research, one contribution of the current findings is the link between synchrony, an experience occurring between two people and not in the individual’s mind, and the shared neural empathy network. Such findings may begin to chart a two-person neuroscience that may afford a new framework for this age-old dilemma.

It is estimated that one in five children world-wide is growing up in the context of continuous war, terrorism, tribal strife, or ethnic and religious struggles^57^ and the long-term impact of such rearing contexts on the developing brain are still unknown. Our study is pioneer in following children growing in a war-zone and measuring brain response to distress at an important developmental transition. Since early adolescence is also the period when anxiety and depressive disorders increase in prevalence^56^, the present findings have important translational implications for tapping the neural mechanisms that may mediate the development of anxiety disorders in war-exposed children. The study fills a gap in research on trauma and its effects on the developing brain by addressing the neural consequences of chronic exposure to war and terror, a condition that impacts millions of children world-wide^57^. Our study also conforms with the call that risk and resilience should be studied longitudinally across lengthy periods of time, begin early^58^, focus on neurobiological outcomes^36^, and uniquely integrate early caregiving in the aftermath of trauma. Much further research is required to follow children exposed to repeated trauma as they grow older, integrate direct observations of the caregiving context over time, and address the combined contributions of biology and parenting to the maturation of the brain basis of affiliative, affective, and socio-cognitive processes.

## Methods

### Participants

Participants were recruited in two groups and observed four times as follows.

At T1 (early childhood), we recruited 232 children (*M* = 2.76 years, SD = 0.91) and their families, including 47.6% males and 47.1% firstborns. The war-exposed group included 148 families living in the same neighborhoods in Sderot, Israel, a small town located 10 km from the Gaza border and exposed to continuous and unpredictable rockets and missiles attack for over 20 years. The control group included 84 nonexposed families from comparable towns in the greater Tel-Aviv area matched to exposed group in age, gender, birth order, parental age and education, maternal employment, and marital status and screened for other trauma.

At T3 (late childhood; *M* = 9.3 years, SD = 1.41), 177 families were revisited with attrition was mainly related to inability to locate families or families moving out of Sdeort. At T4 (preadolescence), 84 children (*M* = 11.81 years, SD = 1.24) participated in MEG scanning, including 49 females, and half of the sample (*n* = 42) were war-exposed. Of 111 children participating in T4, 27 did not complete the MEG experiment due to the following: 18 were MEG-incompatible (mostly due to metals), 6 declined the MEG part, and 3 did not complete the MEG paradigm. Study was approved by the Bar-Ilan University Institutional Review Board and a written informed consent was obtained from parents after receiving a complete description of the study. All experiments were performed in accordance with the relevant guidelines and regulations.

### Procedure and measures

T1 early childhood: during a 3.5-h home visit, 10-min mother-child interaction with age-appropriate toys was filmed. Interactions were coded with the well-validated coding interactive behavior system (CIB)^59^, and the mother–child synchrony construct was used. Child negative reactivity was assessed with a procedure adapted from the well-validated LAB-TAB^60^. Child sat in front of an experimenter, who put on four masks of increasing fearfulness while child was looking: clown, pet animal, scary animal, and ghost. The experimenter put on the mask, called the child’s name, and left it on for 10 s. The fear paradigm was micro-coded offline in 0.01-s frames on a computerized system and the negative reactivity construct was the sum of the following codes: fuss, cry, whine, displaying negative facial expression, and gaze aversion. Two coders coded the fear episode and reliability, measured on 20 interactions, averaged kappa = 0.82 (range: 0.73–0.91).

At T3 (late childhood), children were observed in two mother–child interaction paradigms (positive and conflict) each lasting 7 min and videos were coded offline using the CIB adolescent version, consistent with studies of that age. The mother–child synchrony at both T1 and T3 included the following averaged codes: synchrony, dyadic reciprocity, mutual adaptation/regulation, fluency, and empathic involvement. The final construct was averaged from the interactions at T1 and T3 (alpha = 0.78–0.71). Coding at both T1 and T3 were conducted by trained coders with 20% of tapes coded for reliability. Reliability exceeded 90% on all codes (intraclass *r* = 0.94, range:0.90−0.99). Child anxiety disorders—was diagnosed using the developmental and well-being assessment (DAWBA), a well-validated structured interview generating ICD-10 and DSM-IV psychiatric diagnoses in 5- to 17-year-old children^61^. The DAWBA was administered by clinicians supervised by child psychiatrist, blind to any other information, with reliability >85% and cases conferred every few weeks. Anxiety disorders included generalized anxiety disorder, separation anxiety, panic disorder, specific phobias, and post-traumatic stress disorder.

At T4 (preadolescence), during MEG scanning, we employed an empathy task contrasting situations where same-age targets were in distress (DS) versus nondistress (no-DS) and children were asked to take the targets’ perspective and put themselves in the other person’s shoes^22^. This experimental contrast involves sensitivity to vicarious distress and activates both the affective and cognitive components of empathy^22^. The paradigm also required participants to self-report on two empathy-based aspects: (a) the ease of perspective-taking (how easy was it for you to take the perspective of the protagonist?), tapping into cognitive empathy, and (b) the perceived targets’ emotions (i.e., affective valence, and arousal), which addresses affective empathy.

### Stimuli

We created pool of 128 stimuli (photos in uniform size: 300 × 225 pixels) half depicting distress/anxiety situations and half neutral. Distress situations described typical anxiety-promoting (social exclusion and exam stress), versus nondistressing (shoe-lacing and reading) events in preadolescents’ lives. Stimuli were piloted until the final 128 stimuli were each validated by independent raters (*n* = 21). Stimuli’s affective valence (1-very negative, 2-negative, 3-neutral, 4-positive, and 5-very positive) was rated as neutral (*M* = 3.04, SD = 0.25) and negative (M = 1.95, SD = 0.28) for the no-DS and DS stimuli, respectively, with a statistically significant difference (*P* = 6.21 × 10^−47^) between categories. Stimuli’s affective arousal (1-very low to 5-very high) was rated as low (*M* = 2.05, SD = 0.33) and high (M = 3.83, SD = 0.42) for the no-DS and DS stimuli, respectively, with a statistically significant difference (*P* = 2.37 × 10^−53^) between categories. Finally, stimuli were matched for physical parameters, including complexity, contrast, and luminance, resulting in no statistically significant difference (*P* > 0.35) on any of these parameters.

Photos were presented in blocks preceded by a contextual sentence, generically describing the situation in the ensuing photo (e.g., “this person heard that his friends plan to exclude him”, “this person reads about the history of Sweden”). Sentences were designed to consist of *M* = 9.0, SD = 1.14 words and *M* = 43.64, SD = 5.10 characters long, with no statistically significant difference (*P* > 0.3) in length between categories. Paradigm was programmed and operated using E-Prime^®^ 2 software (Psychology Software Tools Incorporated).

### Imaging session

Participants laid in supine position inside the MEG system while facing a screen projecting the stimuli in the center of gray background of 20-in monitor at distance of 50 cm. Participants were told to take the targets’ perspective and to imagine how he/she felt in that situation. Fourteen blocks consisted each of a contextual sentence describing the situation followed by 8–10 photos depicting different individuals in that situation. Sentences and photos were presented for 10 and 2 s, respectively. The interstimulus interval was jittered for 1.170–2.004 s and the interblock interval was jittered for 4.170–5.004 s. Participants were trained by watching two exemplar blocks and instructed to remain relaxed and not move their head or body and to pay attention to the events depicted in the photos. Movements were visually monitored by the experimenter via a camera, and by a movement-tracking system using five coils attached to the participants’ scalp to record head position relative to the sensor array. Noteworthy, conventional MEG is not ideally suited for children due to the large helmets and to the difficulty for most children to continuously remain focused and still, thereby often resulting in large head-movements^62^. While head-position deviations were on average (*M* = 0.36, SD = 0.28 cm) below the advised threshold of 0.5 cm (i.e., the typical coregistration error^63^), 25% of the preadolescents exceeded the threshold. Yet, because it is very difficult to avoid head-movements in pediatric populations, we reasoned that retaining 25% (i.e., 21 participants) of this precious longitudinal sample is preferable, while taking into account that the precision of the reported sources should be limited to 1 cm, instead of 0.5 cm.

### MEG recordings and data preprocessing

We recorded ongoing brain activity (sampling rate, 1017 Hz, online 1–400 Hz band-pass filter) using a whole-head 248-channel magnetometer array (4-D Neuroimaging, Magnes^®^ 3600 WH) inside magnetically shielded room. Reference coils located approximately 30 cm above the head, oriented by *x*-, *y-*, and *z-*axes enabled removal of environmental noise. Head shape underwent manual digitization (Polhemus FASTRAK^®^ digitizer). External noise (e.g., power-line, mechanical vibrations) and heartbeat artifacts were removed from the data using a predesigned algorithm for that purpose^64^ and trials containing muscle artifacts and signal jumps were rejected from further analysis by visual inspection. We analyzed data of 2000 ms epochs including baseline period of 700 ms filtered in the 1–200 Hz range with 10 s padding and then resampled to 400 Hz.

### MEG analyses

We analyzed data in alignment to stimulus onset and then averaged the power estimates across tapers. We performed analyses using MATLAB 11 (MathWorks^®^, Natick, MA, USA) and the FieldTrip software toolbox^65^. To calculate induced oscillatory activity in the alpha band, a Hanning taper, applied to each epoch of the 248-sensor data yielded the FFT for short sliding time windows of 0.5 s in the 6–15 Hz frequency range, resulting in spectral resolution of 2 Hz. For source localization, we built a single-shell brain model based on MNI postpuberty template brain^66^, modified to fit each subject’s digitized head shape using SPM8 (Wellcome Department of Imaging Neuroscience, University College London,www.fil.ion.ucl.ac.uk). The subject’s brain volume was then divided into a regular grid. The grid positions were obtained by a linear transformation of the grid positions in a canonical 1-cm grid. This procedure facilitates the group analysis because no spatial interpolation of the volumes of reconstructed activity is required. For each grid position, spatial filters were reconstructed in the aim of optimally passing activity from the location of interest, while suppressing activity which was not of interest. The spatial filter which we applied relies on partial canonical correlations^65,67^ and its CSD matrix was computed between all MEG sensor pairs from the Fourier transforms of the tapered data epochs at the statistically significant time-frequency sensor pattern (Fig. 2, left upper panel).

### Mediation and statistics

To test for mediation, we used process modeling outlined by Hayes^68^ using PROCESS macro. Unstandardized indirect effects were computed for each 10,000 bootstrapped samples and the 95% confidence interval was computed by determining the indirect effects at the 2.5th and 97.5th percentiles. Our brain-based statistics relied on a (two-tailed) non-parametric approach^69^, which takes the cross-subject variance into account, as this variance is the basis for width of the randomization distribution and yields a correction for multiple comparisons. This cluster-based procedure allowed us to obtain a correction for multiple comparisons at all sensor and source analyses. At the virtual sensor and behavioral levels, however, such procedure was not possible given the different nature of analyses; therefore, at those levels, all tests were two-sided, and underwent correction for multiple comparisons by controlling the false-discovery rate^70^.

### Reporting summary

Further information on research design is available in the Nature Research Reporting Summary linked to this article.

## Supplementary information

Supplementary Information

Reporting Summary

## Data Availability

The data that support the findings of this study are available on request from the corresponding author. The data are not publicly available due to them containing information that could compromise research participants’ consent.

## Code availability

The study was developed based on Matlab (MathWorks, Inc.) using a freely available toolbox http://www.fieldtriptoolbox.org/ and custom code.
