# Supplementary material for: The neural development of empathy is sensitive to caregiving and early trauma
Source: Nat Commun. 2019 Apr 23;10:1905. doi: 10.1038/s41467-019-09927-y (PMC6478745; doi:10.1038/s41467-019-09927-y)
Supplement: Supplementary file 1 — Supplementary Information [file 41467_2019_9927_MOESM1_ESM.pdf]

Supplementary Information

Contents: Supplementary Table 1

Supplementary Table 1. Pearson r correlation between networks' activation and other variables

	Empathy Response		Mentalizing / Reactivity	
Variables	r	p	r	p
Synchrony	.34	.002**	-.10	.34
Reactivity	-.12	.27	.25	.03*
Empathy – perspective-taking	.05	.62	.005	.97
Empathy – arousal	-.16	.15	-.002	.99
Empathy – valence	.19	.09	.17	.14

^ The variables in both time points (T1 and T3) were highly correlated to each other ($r = .60$, $p < .0000001$).

** $P_{\text{FDR-corrected}} < 0.05$

* $P_{\text{FDR-uncorrected}} < 0.$
